# Is Health Related Quality of Life (HRQoL) a valid indicator for health systems evaluation?

**DOI:** 10.1186/2193-1801-2-664

**Published:** 2013-12-11

**Authors:** Martin Romero, David Vivas-Consuelo, Nelson Alvis-Guzman

**Affiliations:** Fundación Salutia, Centro de investigación en Salud, Bogotá, Colombia; Doctarado en Salud Publica, Universidad Nacional de Colombia, Bogotá, Colombia; Research Center for Health Economics and Management, Universitat Politècnica de València, Valencia, Spain; Research Group on Health Economics, Universidad de Cartagena, Cartagena, Colombia; Research and Teaching Center, Hospital Infantil Napoleón Franco Pareja, Cartagena, Colombia

**Keywords:** Quality of life, Quality of Healthcare public health, Life Expectancies

## Abstract

The purpose of this review is to do a discussion about the use of the HRQoL as a health measure of the populations that enable to analyze its potential use as a measure of development and efficiency of health systems. The principal use of the HRQoL is in health technologies economics evaluation; however this measure can be use in public health when need to know the health state of population. The WHO recognizes its potential use but its necessary to do a discussion about your difficulties for its application and restrictions for its use as a performance indicator for the health systems.

The review show the different aspects about the use of HRQoL how a measure of efficiency ot the health system, each aspect identified in the literature is analyzed and discussed, developing the pros and cons of their possible use, especially when it comes as a cardinal measure.

The analysis allows recognize that measuring HRQoL in countries could serve as a useful indicator, especially when it seeks to measure the level of health and disease, as do most of the indicators of current use. However, the methodological constraints that do not allow comparability between countries especially when you have large socioeconomic differences have yet to be resolved to allow comparison between different regions.

**Electronic supplementary material:**

The online version of this article (doi:10.1186/2193-1801-2-664) contains supplementary material, which is available to authorized users.

## Introduction

Health Related Quality of Life (HRQoL), as a measurement of the health status of individuals, was first used by the second half of the twentieth century, although its greater use was observed since the end of the last century, using the estimate of Quality Adjusted Life Years (QALY), as an outcomes measurement for the economic evaluation of healthcare technologies. QALYs are understood as a comprehensive measure of the health state of an individual and which corresponds to the result of a composite function, on one hand by the HRQoL measurement (subjective measure) using a cardinal scale between 0 and 1, and by the amount of life years (objective measure) on the other hand. Thus, it seeks to establish, in a single value, the health state of an individual regarding a health or disease moment in time so that one “1” would be equivalent to a perfect state of health and zero “0” would be equivalent to being dead, with the possibility of an individual having results worse than dead, meaning, negative results. Although the main use of the QALY has been in economic evaluations as an outcome measurement of the cost-utility analysis, it has been proposed as an indicator that reveals changes, not individually but collectively and additively, in the health state of a population and that, therefore, reflects the impact generated by a healthcare system (PATRICK and Erickson 1993; WHO 2003).

The objective of this essay is to explore the use of the HRQoL as a health measure of the populations and its potential as an outcome measure for the performance evaluation of the health systems and/or the actions in public health. In its development we review several proposals that pose its use, the technical and/or methodological difficulties for its application and, finally, we discuss about the difficulties or restrictions for its use as a performance indicator for the health systems.

### HRQoL background

There is no consensus regarding a definition of Quality of Life, and when we think about it, we usually fall into defining it around itself or into the description of the integrating aspects. However, as suggested by Fayers and Machin, generally the western individuals have a similar conception and most relate it to the fact of reaching happiness and satisfaction in life (Fayers and Machin 2007). This means, the quality of life will always be related to the situation perceived by the individuals according to the environment where they develop.

One of the first mentions to the subject of Quality of Life and its relationship with health can be found in a document by Aristotle; in its Nicomachean Ethics he refers to the harmony obtained in the *good life* as sense of happiness, which is valued by the people depending on the moment in which they are, “when you are sick it is obtained with the health or when you are poor it is obtained with richness” (Aristotles 1999). Thus, although health seems inherent to the concept of quality of life, when we want to mention health specifically, moving away from the concept of quality of life – used to establish the development level of a population - little progress has been made since the initial idea of Aristotle. Only at the middle of the XX century the subject of health, related to quality of life begins to be mentioned, when the World Health Organization (WHO) on its 1946 assembly adopts the definition of health as “a state of complete physical, mental and social well-being and not merely the absence of disease or infirmity” (OMS 1948)*.* This fact marks a milestone in relating the health with the quality of life, giving rise to a different category which is mentioned as the Health Related Quality of Life, HRQoL, different from the general measure of quality of life and directly associated to a health state of an individual, and on this we base our discussion.

Several definitions of HRQoL precede the definition of HRQoL proposed by the WHO (1997); an “individuals perception of their position in life in the context of the culture and value systems in which they live and in relation to their goals, expectations, standards and concerns” (WHO 1997). For example: Patrick and Erickson (1993) define it as: *“The measure in which the assigned value is modified to the duration of the life in function of the perception of physic, psychological and social limitations and the decrease of opportunities due to the disease, its sequels, the treatment and/or the health policies”* (PATRICK and Erickson 1993); or Schumaker and Naughton (1996) as, *“a subjective perception, influenced by the current health status, of the ability to perform those activities important for the individual”* (NAUGHTON et al. 1996). Despite the different approaches in the definition, it is evidenced that it is based in a perception by the individual around his well-being, which is of a multidimensional order and includes the current health situation, not the disease, and his gaze into the future.

### HRQoL approches

Although there are different approaches to the measurement of the HRQoL, the necessity of understanding it as an indicator of development bring us facing the concepts of cardinal measurement. That is to say, to have a specific and continuous number that allows giving a value to the health status of an individual and that it could be added around a social group. The concept of quality of life is developed based on the Welfare Theory of Bentham and followers; assuming that the goal of the social action is to promote the maximum happiness for most people, understood as the maximum welfare for some and the least damage for others. However, the concept of cardinality of the measurement of quality of life, is based on the application of the Theory of Expected Utility, proposed by J. von Neumann and O. Morgenstern (Von Neumann and Morgenstern 1967). In this it is proposed that individuals have a basket of goods on which they can make a rational choice under uncertainty, such they have the ability to pick and complement their choices, based on three basic axioms: a) all alternatives are comparable (completeness), b) the preferences of individuals do not change abruptly (continuity) and c) transitivity exists between the different alternatives. It is assumed that health is a sequence of future states identified by the individual and that all individuals are faced with a finite set of possible health outcomes that behave as lotteries in time (Torrance 1976). Accordingly, it is proposed that it is possible to measure the level of health loss of an individual based on the compensation that he might choose for his possessions; in this case, in exchange for the years to live. This choice is made by the individual and is based on uncertainty, as in Standard Gamble (SG) and in the Time Trade-Off (TTO) (Wright et al. 2009).

### Measuring overall health systems performance

**Figure 1:**
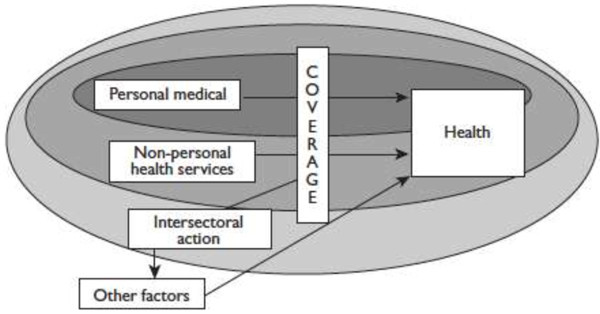
**Defining health system by components.**

In order to improve or maintain the health of individuals and the community, states establish institutional arrangements called Health Systems. As WHO states, there are different definitions of a health system, depending on the scope thereof, (different ovals in Figure 1) but definitely all correspond to a single primary objective which is to improve and protect the health (WHO 2003). Consequently, the question that concerns us here is aimed at determining, what would be the measures that allow us to evaluate the outcome of the Health Systems in its ultimate goal (health)?

Taken from: Murray, C. J., and Evans, D. B. (Eds.). (2003). *Health systems performance assessment debates, methods and empiricism.* Geneva: World Health Organization.

Because of the health report of 2000, and the increasingly frequent reforms of health systems, WHO convened a group of international experts to discuss a proposal that would analyze the performance of health systems, whose results were compiled, although not unanimous, in *Health systems performance assessment debates, methods and empiricism* (WHO 2003) focusing its analysis on five measures: *health, health coverage, level of offer, population coverage and equity financing.* Thus, arises that efficiency of a health system only can be understood as a reason that compares the results obtained and the level of resources allocated to health (Tandon et al. 2003) and then it is suggested that the assessment should be based on an economic analysis that compares as an *input* the health expenditure and its distribution and as an *output* of the specific health outcomes or preferably the valuing a global measure of health (Evans et al. 2003). Being the latter aspect the element of discussion in this essay.

### Life expectancy and mortality indicators as health indicators at the present time

Since 1995, WHO publishes the world health report whose main objective is the comparative consolidation of information about the health status of populations across the different countries and health systems. Each report, additional to the consolidation of indicators, deepens on a central topic on which a specific analysis is done. Among the different indicators used in these reports, the measures related to disease and mortality are the most used. However, from the year 2000 there are new measures based on the well-being, by showing the Disability Adjusted Life Expectancy (DALE) as a measure of disease burden in different countries. And, since 2001, we observe different HRQoL annotations when analyzing specific pathologies in such reports. In that sense, when reviewing the indicators that describe the health status of populations, life expectancy at birth, life expectancy at 60 years and infant and maternal mortality have been most commonly used to show, objectively, changes within and between countries. Life expectancy was proposed, since the 60s, as an overall indicator to give a proper health measure and transcend the description of morbidity and mortality (Sanders 1964). In the United States, life expectancy at birth in the early twentieth century was 47.3 years and in 2007 was 77.9 years (Molla et al. 2003a), in Colombia was 48.3 years in 1950 and moved to 73.5 in 2010. This indicator improved dramatically during the twentieth century, especially due to the control of infectious diseases and major changes in health conditions. And, although there are still differences between countries, in general, changes from year to year are dwindling, as are the differences. This problem of loss of sensitivity of the indicator is noteworthy, given the epidemiological transition in both developed and developing countries. The problem with this indicator is that it is not sensitive to the aging of the current population or to the presence of chronic diseases, which instead of affecting mortality, affects the quality of life of the population (Molla et al. 2003b).

In 2010 Chen and Mahal propose replacing traditional measures by a measure based on quality of life and disability taking into account the new characteristics of the population (Chen and Mahal 2010). Some authors have shown how changes in the population pyramid due to the chronicity of diseases (Acemoglu and Johnson 2007; Cervellati and Sunde 2009), or lowering fertility rates (Cervellati and Sunde 2011), have affected life expectancy without being a true effect of development.

The Institute of Medicine (1998) revised the meaning of the indicators that measure the health and concluded about the great limiting than implies to continue using indicators based on mortality, inasmuch as they do not reflect changes in the health of the current population, proposing the use of composite indicators that blend both effects such as death and disease through a measure of HRQoL and QALY (Institute of Medicine, National Academy of Science 1998).

### Do classic indicators (mortality/illness) allow the health systems performance assessment?

Facing the main objective of the health systems of improving or maintaining the health of individuals and populations, a limiting of the classic indicators is that its measure is based in mortality (Gulis 2000). In that sense, Jeremic et al. (Jeremic et al. 2011) claim that measurements based on mortality, correspond to a partial view that does not holistically embrace the concept of health and generate constraints when analyzing health systems, although argue that they are still used as a valid element due to its high objectivity based on certain data (deaths). In a critical review, in 2006, it is shown how most authors when referring to measure the impact on public health, restrict themselves to the results in deaths, life expectancy and that only some go further to include the disease burden (Thacker et al. 2006).

On the other hand, Frenk and Murray (2000) had already suggested that the outcomes measures of health systems, should reflect what is happening throughout life, understanding the growing criticism against the measures of life expectancy, but assuming that it was sufficient to add the equity analysis as an element of complement (Murray and Frenk 2000). However, it is in 2003 when WHO (WHO 2003) proposes to take the average of HRQoL as a direct measure that allows the assessment of the efficiency of the systems. Joshua A Salomon, et al. (Salomon et al. 2003) discuss the importance of measuring a spectrum based on health states that move between perfect health and death, such as raised from the measurement of HRQOL, applying multidimensional measurement schemes, based on preferences or utilities, thus assuming that in this way the health of people is measured in a real form. Although they recognize a critical fact when facing the decision of taking these new indicators, which is the limiting of information.

It is evident then, that the use of indicators based solely on mortality to assess the health status of populations with high proportions of people older than 60 years and a high prevalence of chronic diseases, poses serious limitations to measure the performance of health systems. It is therefore necessary to have indicators to assess health as the objective of the systems – and not the disease - for which HRQoL measurement would assess the performance of health systems.

### HRQoL measurement as health sysmems key performance indicator

There are two main approaches to measuring HRQoL: state measures by themselves and those based on giving a "value" to the HRQoL (Wright et al. 2009) in an orderly manner and have resorted to economic theory as a means of development. Although there is no widely dominant or hegemonic model, when we refer to measurement schemes that somehow beyond a relative order quantify and allow comparability, is the economic theory that has given an explanatory approach. Thus, depending on the method of collecting HRQoL data, two methodologies are identified: (i) direct measurement of preference choices and (ii) preferences based on health status classification systems by multidimensional analysis (McDonough and Tosteson 2007).

Direct methods try to identify an individual´s preference regarding a single attribute. The Visual Analog Scale (VAS), Standard Gamble (SG) and Time Trade-Off (TTO) techniques are identified under this method. Preferences classification systems, based on health status, rely on the measurement of different characteristics called *domains* which seek an approximate quantification of the quality of life (McDonough and Tosteson 2007; Prieto and Sacristan 2003).

The VAS has been used to measure HRQoL in some pathologies such as depression (Mykletun et al. 2001; Cleland et al. 2007), multiple sclerosis (Poole and Steen 1991), pain (Poole and Steen 1991), rheumatic disease (Poole and Steen 1991) and in addition to some multidimensional scaling. It was used in the measurement proposed by the WHO report (2000), assessing different dimensions and applying it in several countries including Colombia (Salomon et al. 2003). Despite its ease of use, the main discussion of the VAS is in its construction itself, because it assumes that the individual is able to accurately quantify health and give an accurate measure of loss of health, as if he could assess the loss of quality of life persistently and consistently over time.

The other two methods (SG and TTO) are based on different approaches of the concepts of Expected Utility Theory (Von Neumann and Morgenstern 1967). They start from the premise that health is an important argument in the utility function of individuals, in such a manner that we could measure changes in well being associated with a loss of health, if we can determine the compensation with the other arguments persisting in a measure of the unchanged utility function. In the SG, lost health is measured by the level of risk that an individual is willing to take, and the utility is evaluated as a negative function of such risk. In the TTO utility is measured by the amount of life expectancy an individual is willing to lose, understanding the utility as a positive function around longevity (Dolan et al. 1996). These last two methods are the choice when validating one of the other mentioned methods is required.

Multidimensional measurement schemes aim to identify the categories or characteristics, which to an individual could correspond to a component of what would mean the quality of life. As stated by Salomon et al. (Salomon et al. 2003), multidimensionality favors the acceptance of the possibility of obtaining a cardinal measure, proposing those basic characteristics that would exist in a measurement. Based on this method, a number of oriented scales have been developed, depending on their use, into two major types: (i) of general purpose, which summarize health states of communities and (ii) the specific for each type of disease. The first, very useful to establish comparability between diseases and overall results of the country, and the latter for specific analysis and as a follow-up support to clinimetry in patients. The most commonly used general measurement systems include EQ-5D (Roset et al. 1999; Krabbe et al. 2004) recently EQ-5D5L (Herdman et al. 2011), the Health Utilities Index (HUI) (Horsman et al. 2003), the Quality of Well Being (QWB) (Pyne et al. 2003) and the SF-6D (Konerding et al. 2009) which was derived from SF-36 (Ware and Sherbourne 1992; McHorney et al. 1993; McHorney et al. 1994).

### The use of HRQOL as a measure of performance of health systems

As mentioned by Neumann et al. (Neumann et al. 2008), there is still a gap between what researchers, especially economists, have considered as the measuring element of efficiency of public health programs and what service providers consider. Assessments of cost-benefit, cost-effectiveness and specially cost-utility are predominant for the first, while the latter base their considerations on non-clustered mediate results and occasionally around life expectancy. However, there is a tendency to include the measurement of quality of life as a reflection of health status, as an approximation of population status even comparatively between countries (Wang et al. 2005). Latin America is also growing in these experiences according to available reports of Argentina (Augustovski et al. 2009) and Chile (Zarate et al. 2011). Although in Colombia already exists a large number of studies on HRQOL conducted on diseases, there is no evidence of a global measure of the population. Although it is argued that the main constraint against its use, as an indicator of global use, is based on the technical difficulties of applying different methods and the lack of current information that allows us to establish the measures, that the discussion should revolve around issues that have not yet been fully resolved (Konerding et al. 2009).

For example, restrictions on its coverage for the entire life cycle, based on the questions to measurement in children (Vogels et al. 1998), or people with major mental disability (Cook and Harman 2008) that have limited its use almost exclusively to the adult population, or to the acceptance of the information obtained from caregivers. But the most critical aspect to be solved, in our opinion, is based on the limitations of using the results transnationally. Bernet et al. applied EQ-5D in Spain, the Netherlands and Germany and found that differences by socio-demographic factors such as education, marital status or income could be the origin of the variations in the measurements; although none of those factors individually represented a difference enough to be considered preponderant (Bernert et al. 2009). In this regard, several studies have recognized differences in HRQoL measures, due to socio-economic factors such as location (rural or urban) (le Hoi et al. 2010), gender and age (Tajvar et al. 2008), religious, cultural (Chatters 2000) and racial differences (Pereira et al. 2011). And according to Prause et al. married people perceive better HRQoL than the divorced, and those living in rural areas better than those in big cities, except if they belong to very high socioeconomic levels and in general women have a worse perception of quality of life than men (Prause et al. 2005). In Colombia, a study oriented to another analysis showed how socio-economic factors affected the quality of life of 512 older adults (Paternina and Melguizo 2010). Thus, it is possible to understand how factors related to the social environment and personal development are important in the perception that leads to the measurement; so that these variations in populations would make the comparability difficult, regardless of results.

Current studies when referring to differences between measurements are restricted to describe the characteristics of populations, and the explanation of these differences has deepened very little. In 2009 a study was conducted with 21,590 adolescents in 13 European countries, using a multilevel analysis to explain the differences by age, gender, and country where the study was conducted. This study shows how changes in country (top level) affected the final result. However, they did not analyze the differences between social classes according to age and gender (Anderson et al. 2010). Jia et al. (Jia et al. 2009) from a sample of patients surveyed between 1999 and 2001 using the Behavioral Risk Factor Surveillance System, showed differences in lost days of mental and physical health, related to socio-economic differences between territories in the United States, using a multilevel analysis. These first approaches open the possibility of recurring in a comprehensive manner to the multilevel analysis as a mechanism of explanation and probably of comparison between countries and even regions.

In this way, if we can establish the existing relations between different levels of analysis and if we can identify the same between the segments to compare, probably we can progress in the analysis. Likewise, the measurement of HRQoL could be an indicator that would allow the reflection of the health status of a population and could be useful as a measure of performance of health systems. However, it would be necessary to count on utility-based measures (cardinal) that allow formalizing the differences both in time as well as between countries. And so, develop multilevel models that incorporate the individual dimension (first level), the family environment (second level) and the regional and/or cultural environment (third level) to clear the way in order to evidence differences regarding the health status, by neutralizing the differences explained by other factors. This opens a new space for the use of HRQoL measures as a valid option to conduct transnational comparisons and even of population segments between countries. In this sense, in countries with large socio-economic differences and high levels of inequality and inequity, like Latin American countries, it would be useful to consider this new type of analysis, in order to have, from the conducted measurements, results useful in time and comparable between health systems and between countries.

## Authors’ original submitted files for images

Below are the links to the authors’ original submitted files for images. Authors’ original file for figure 1
