# Supplementary figures and images for: Is Health Related Quality of Life (HRQoL) a valid indicator for health systems evaluation?
Source: Springerplus. 2013 Dec 11;2(1):664. doi: 10.1186/2193-1801-2-664 (PMC3866375; doi:10.1186/2193-1801-2-664)

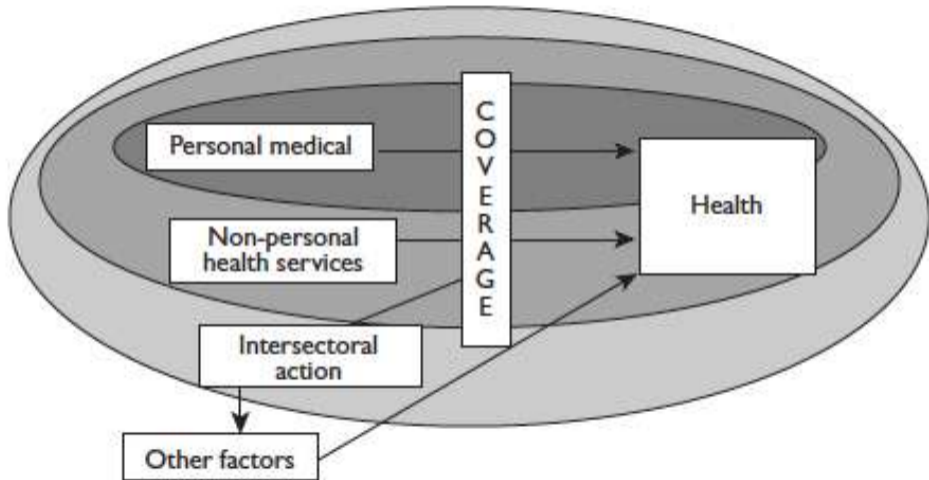

Supplement: Supplementary file 1 — Authors’ original file for figure 1 [file 40064_2013_731_MOESM1_ESM.pdf]
